# Development and Validation of an HPLC-DAD Method for the Quantitative Determination of Benzoyl Peroxide, Curcumin, Rosmarinic Acid, Resveratrol and Salicylic Acid in a Face Mask—In Vitro/Ex Vivo Permeability Study

**DOI:** 10.3390/molecules30224474

**Published:** 2025-11-19

**Authors:** Sofia Almpani, Maria Mitsiou, Paraskevi Kyriaki Monou, Catherine K. Markopoulou

**Affiliations:** 1 Laboratory of Pharmaceutical Analysis, Department of Pharmacy, Aristotle University of Thessaloniki, 54124 Thessaloniki, Greece; 2 Laboratory of Pharmaceutical Technology, Department of Pharmacy, Aristotle University of Thessaloniki, 54124 Thessaloniki, Greece

**Keywords:** HPLC-DAD, benzoyl peroxide, salicylic acid, curcumin, rosmarinic acid and resveratrol, anti-acne facial mask, experimental design, in vitro/ex vivo permeability study

## Abstract

Acne is a common skin condition that causes pimples, redness and inflammation. Benzoyl peroxide (BENZ), salicylic acid (SAL), curcumin (CUR), rosmarinic acid (ROS) and resveratrol (RESV) exhibit antimicrobial, anti-inflammatory and antioxidant properties and are recommended for its treatment. These five active pharmaceutical ingredients (APIs) were incorporated into a green clay, honey and gelatin face mask and determined by an HPLC-DAD (diode array) method. For the chromatographic separation of the analytes, a gradient mobile phase with two solvents mixtures: A, comprising H_2_O with 0.1% TFA-ACN with 0.1% TFA, 85:15 *v*/*v*, and B, comprising 100% ACN with 0.1% TFA, and a C18 column (250 × 4.6 mm, 5 μm), at 40 °C (diluent: MeOH-ACN 0.1% TFA 2:1 *v*/*v*), were selected. The method was validated according to the ICH guidelines for pharmaceutical products (R^2^ > 0.999, %RSD < 1.2, % Recovery > 98.2, LOD_μg/mL_: ROS = 0.267, RES = 0.047, SAL = 0.636, CUR = 0.296 and BENZ = 0.083). For the processing of mask samples and the quantitative extraction of the analytes, the “D-optima mixture” experimental design methodology was applied (% Recovery 95.4–102.1%, %RSD < 2.4). Finally, the permeability rate (Papp) of the mask ingredients through the skin was studied using Franz vertical diffusion cells, in a cellulose membrane (in vitro), in rat tissue and in human skin (ex vivo). To ensure the reliability of the results, APIs’ stability rate under the given experimental conditions was studied. In addition, a second method for sample processing in Franz cells was developed and validated (% Recovery > 90.6–106.9, %RSD < 5.2). Based on the results obtained, both the effectiveness of the new face mask formulation and the suitability of the membranes were evaluated.

## 1. Introduction

Acne is one of the most common skin diseases (9.4% of the world’s population) that occur especially at a young age and can seriously affect the psychology of adolescents. It is mainly due to the blockage of hair follicles by sebum or dead skin cells, resulting in an alteration of the keratinization process, high production of certain strains of the Cutibacterium bacteria and the creation of inflammation [1]. These symptoms usually appear during a person’s adolescence, when significant hormonal changes occur in their body [2,3].

The healing of the skin in the affected area is difficult, and, usually, after the end of acne, scars remain. For this reason, the pharmaceutical industry focused on the development of cosmetic and herbal products to address the problem. Therapeutic approaches so far are classified into systemic administration preparations and those for topical application [1]. Dermo-cosmetic products include masks, gels and creams that contain antioxidant, healing, antibacterial ingredients such as benzoyl peroxide, salicylic acid, curcumin, rosmarinic acid and resveratrol. Many of these are derived from plant extracts and target one or more of the pathogeneses of acne. Benzoyl peroxide (BENZ) is one of the safest and most effective options, with strong anti-inflammatory healing and antimicrobial effects against many bacteria, including C. acne and fungi, while also helping to normalize the keratinization process [4,5,6]. Resveratrol (RES) may also slow down the skin aging process due to its role as a sirtuin activator (regeneration and cellular enzyme) [7], exhibiting antibacterial, antiviral, antifungal and anti-inflammatory properties [8]. Similarly, salicylic acid (SAL) is a relatively fat-soluble phenolic aromatic acid with anti-inflammatory, antimicrobial and sunscreen properties, which is used topically for skin conditions, including warts, dyschromia, hyperkeratosis and acne [9]. It is commonly found in exfoliating preparations that remove dead keratinocytes from the skin surface and hair follicles, and enhance collagen production. Rosmarinic acid (ROS) is a plant-derived ester of caffeic and 3–4 dihydroxy phenylacetic acid and was first chemically prepared in 1991 by Albrecht [10]. Its strong antioxidant, anti-inflammatory and antiviral properties make ROS an ideal choice for use in cosmetic products [11,12]. At the same time, many studies indicate its effective action against Gram +/− bacteria, both in vitro and in vivo experiments. Finally, curcumin (CUR) is a relatively lipophilic polyphenol with a characteristic bright yellow color obtained from the rhizome of the homonymous plant *Curcuma longa*. It is an ideal ingredient for topical formulations, since it has various beneficial antioxidant, anti-inflammatory and healing properties [13]. It is often used as a monotherapy or in combination with other agents to combat common skin microbes, such as staphylococcus, bacteria and fungi [14,15].

Face masks are products that are easy to apply, act immediately and are removed approximately 20 min to an hour later. They adhere to the surface of the skin to remove dirt, oil, microorganisms and dead cells, while at the same time nourishing it and adding shine. Their ingredients are usually of plant/natural origin such as vitamins, proteins, minerals, honey, clay, etc. Depending on their texture and method of application, they are divided into four categories: (a) Sheet, (b) Rinse-off, (c) Peel-out and (d) Hydrogel masks [16].

In order for a transdermal formulation to be approved by the competent authorities, relevant skin permeability studies of APIs in vitro and ex vivo must be conducted [17,18]. In vivo studies for animals are rare, as these models require additional time and cost, and are subject to bioethical restrictions [19]. Ex vivo experiments are usually performed on human or animal tissues (e.g., rats, guinea pigs, rabbits, monkeys, cows and sheep) [20,21]. However, when such tissues are not readily available, synthetic membranes (in vitro) are recommended by the Food and Drug Administration (FDA) [22]. Accordingly, to carry out such studies, a vertical Franz cell diffusion device is usually suggested, which functions as simulator [23], providing basic information about drugs behavior [24].

In general, there are many reports of analytical methods for the determination of the five antioxidants in various substrates. HPLC-UV chromatography with a stationary phase C18 column, of different dimensions (150 or 250 × 4.6 mm, 5 μm) and a mobile phase usually consisting of acidic aqueous solutions (phosphate or ammonium acetate buffer pH < 5, 0.1% trifluoroacetic acid, 0.1% acetic acid and phosphoric acid) and acetonitrile or methanol is mainly proposed. SAL has been determined in human saliva and plasma [25], in cosmetics with benzoyl acid [26], in analgesic drugs (topical application) [27] or in pure raw materials [28]. Salicylic acid has also been determined as the sole component in flour [29] or in the presence of other analytes such as tretinoin (in skin preparations) [30] or clindamycin (in gels) [31]. The reverse-phase HPLC-UV method is also used to detect CUR in turmeric extract [32] or in rat plasma [33]. Similarly, chromatographic methods have been reported for the determination of ROS (in Salvia officinalis plants) [34], alone or in combination with caffeic acid (in aromatic plants and pharmaceutical preparations) [35] or with carnosic acid and carnosol (in rosemary extract) [36]. Finally, ROS, chlorogenic, rosmarinic and caffeic acids were determined simultaneously in medicinal plants by HPLC [37]. Resveratrol is usually determined by HPLC with UV or fluorescence detector, or with gas chromatography [38]. C18 columns are commonly suggested as stationary phase, whereas in some cases C8 is also recommended [39]. Wang et al. determined RESV in a w/o/w emulsion [40] while Scalia et al. prepared a cream (w/o) containing resveratrol in combination with lipid microparticles, and analyzed them with a C18 column and a mobile phase of methanol–water with 0.1% acetic acid (65:35, *v*/*v*) [41]. Τhe determination of resveratrol in the presence of four lipophilic APIs (ferulic acid, quercetin, retinol and α-tocopherol) in a cold cream (w/o) was also analyzed using a reverse-phase HPLC-PDA method [42].

In the present study, five active substances (salicylic acid, benzoic acid peroxide, rosmarinic acid, resveratrol and curcumin) that exhibit antioxidant, regenerative and healing properties were selected, and a face mask was prepared. This composition is indicated for the treatment of acne and consists of green clay, gelatin and honey (excipients). To confirm the quality and integrity of the proposed mask, a new reverse-phase chromatographic HPLC-PDA method was developed and validated, and the five APIs were determined. For the purification of the samples, it was considered necessary to study the stability of the analytes under various experimental conditions. Their extraction was then carried out using an experimental design software and the D-optimal methodology. Finally, the proposed analytical method was applied to study the permeability (Papp) of APIs (in vitro and ex vivo), through artificial cellulose membranes and rat or human tissues. Based on the release rate of the active ingredients and their Papp values, the therapeutic potential and efficacy of the formulation can be evaluated.

## 2. Results and Discussion

### 2.1. Optimization of Chromatographic Method

Due to the similar chemical structure (Supplementary Figure S1) and hydrophobicity of the analytes (Supplementary Table S1), their peaks showed almost identical chromatographic behavior and insufficient separations. To solve the problem, various combinations of stationary and mobile phases were tested. Initially, a C4 and a phenyl ACE^®^ (150 mm × 4.6 mm, 5 µm) column were used as the stationary phase and as the mobile phase methanol with water, in various ratios (with or without 0.2% formic acid or 50 mM phosphate buffer, pH 6.5). However, the separations were not sufficient, the quality of the peaks was not good and the elution of the substances was either with the solvent front (first peak) or over a very long time. To increase the retention of the substances and achieve better separations, reversed-phase columns operating with different retention mechanisms, such as Cyano, Waters Spherisorb^®^ (250 mm × 4.6 mm, 5 µm), were also tested. Acetonitrile with aqueous solutions of 0.2% formic acid or 20 mM phosphate buffers (pH 2.5 or pH 6.4) were used as the mobile phase. To improve the chromatograms, both isocratic and gradient elution were applied, but the results were not satisfactory because although the retention of the first peaks increased, their separation and quality were not good. A hydrophilic interaction column, SeQuant ZIC^®^ HILIC (150 × 4.6 mm, 5 μm), with mobile phases (gradient or isocratic elution) consisting of acetonitrile and buffer solutions (20 mM phosphate at pH 2.5 or 20 mM ammonium acetate at pH 5.5 or 0.2% aqueous formic acid) was also examined. The column improved the shape of the chromatographic peaks but gave insufficient separations. The best chromatographic performance was demonstrated by a reversed-phase column Discovery HS C18 (25 cm × 4.6 mm), 5 μm at 40 °C, which was used for further investigations.

For the composition of the mobile phase in terms of the organic solvent, between methanol and acetonitrile, the latter gave better results and was considered the solvent of choice. Also, the addition of buffers or salt to the water was deemed necessary to improve the quality of the peaks. Regarding the pH value, this could affect the ionization and therefore the retention time only of ROS (pk_acid_ = 4.50) and SAL (pk_acid_ = 2.97), while the rest analytes were not affected (Supplementary Table S1).

Therefore, the use of buffer solutions with ammonium or phosphate salts (5 to 50 mM) allowed the adjustment and investigation of the mobile phase over a wide range of pH values. In the pH range of 5–6.5, where the two acids remain ionized, although the quality of the peaks was relatively better, their separation was poor, and the elution of ROS approached the solvent front. Correspondingly, adjusting the pH between 2.5 and 3.5 maintained all analytes in a non-ionized form, ensuring a better value for the k′ (capacity coefficient) of ROS, while at the same time giving good separations and chromatographic peaks. Low pH values were also achieved by the addition of 0.1% or 0.2% formic acid or with 0.1% trifluoroacetic acid (TFA) or 0.1% acetic acid.

TFA (pKa = 0.23) lowers the pH well below the pKa of weakly acidic compounds, suppressing their ionization. Thus, it improves the tailing factor of acidic moieties such as ROS (pKacidic = 4.5) and SAL (pKacidic 2.97). This ability is usually not sufficient when formic acid (pKa = 3.75) or acetic acid (pKa = 4.76) are used. Thus, among these modifiers, the aqueous solution of 0.1% trifluoroacetic acid was the best choice and participated in the following mobile phase: 0.1% TFA—acetonitrile with 0.1% TFA in a ratio of 85:15 *v*/*v* (phase A)—and 100% acetonitrile with 0.1% TFA (phase B) in a gradient elution system (Table 1).

**Figure 1:**
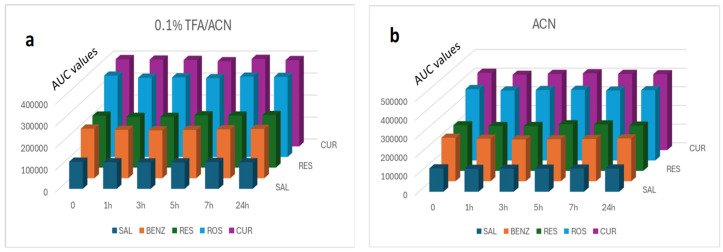
Stability study of analytes in (**a**) ACN 0.1% TFA and (**b**) ACN, at room temperature.

An equally important factor affecting the peak shape was the diluent added to the final standard and sample solutions. The selected solvent should completely dissolve the analytes, be compatible with the mobile phase and give good and reproducible chromatographic peaks. Methanol is considered the solvent of choice because it has the greatest solubilizing capacity for all analytes and ensures their stability. Therefore, its presence is essential both for the preparation of the initial standard solutions and in the final diluted samples. However, because it often does not give good chromatographic peaks, the composition of the diluent needs further investigation. Therefore, methanol was tested alone or with acetonitrile in various ratios with/without 0.1% formic or acetic or trifluoroacetic acid. Also, the same organic solvents were tested in combination with aqueous buffers in various ratios (100% to 50%). Better results were obtained with methanol–acetonitrile in a ratio of 1:1 to 2:1 *v*/*v* or methanol–acetonitrile with 0.1% TFA in a ratio of 1:1 to 2:1 *v*/*v*. In order to select the best diluent, the stability of the analytes at ambient temperature, using ACN or ACN with 0.1%TFA, was checked for a period of 24 h (Figure 1a,b). Based on the results, the samples were stable in all cases. Ultimately, MeOH–acetonitrile with 0.1% TFA 2:1 *v*/*v* was chosen, as it gave slightly better peak shapes.

For the UV detection of APIs, three different wavelengths were used. According to their spectra, λ was set at 320 nm for ROS, RES and SAL, at 234 nm for BENZ and at 400 nm for CUR (Supplementary Figure S2).

### 2.2. Method Validation

According to the International Conference on Harmonization guidelines ICH Q2(R1), the validation of an analytical method is the process of confirming that the method used for a specific analysis is suitable for its intended use and supports the identity, purity, quality and potency of the API and the preparation [43]. Key parameters include the method’s specificity, linearity, repeatability and accuracy.

#### 2.2.1. System Suitability

Part of the validation of an analytical method is the system suitability process, prior to analysis. It is based on the principle that the equipment, analytical procedures and samples constitute a complete system that can be evaluated and that is adequate for the intended analysis. The basic parameters that are calculated and evaluated are the retention time (tr), the retention factor (K′), the resolving power (Rs), the number of theoretical plates (N), the height of the theoretical plates (H) and the peak symmetry factor (As) (Table 2).

#### 2.2.2. Selectivity

**Figure 2:**
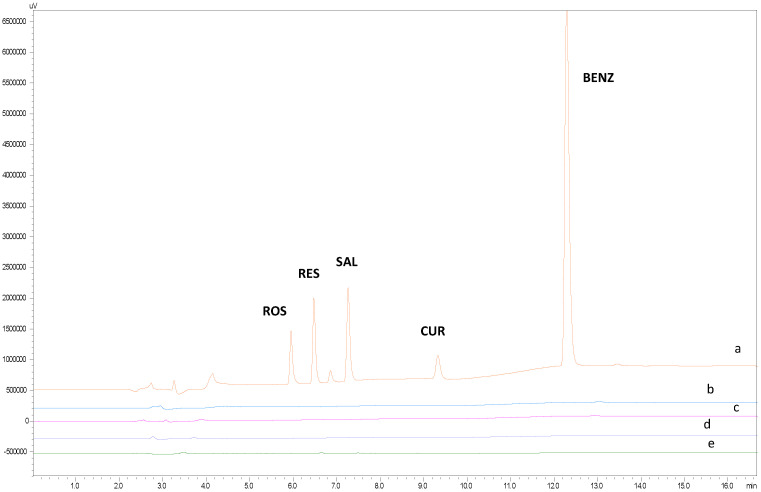
Characteristic chromatograms of (a) standard solution of SAL, BENZ, RES, ROS and CUR (b) blank with diluent, (c) blank with PBS, (d) blank of mask matrix, and (e) blank of tissue.

In practice, selectivity can be verified by spiking appropriate levels of analyte and demonstrating that the assay result is not affected by the presence of the matrix. It is indirectly determined whether any contaminating peaks in the chromatogram affect the peaks of the analytes. Therefore, to test the selectivity of the method (a) a mixed standard solution in diluent (MeOH-ACN with 0.1% TFA 2:1 *v*/*v*), (b) a blank solution with diluent (MeOH-ACN with 0.1% TFA 2:1 *v*/*v*), (c) a blank solution with PBS, (d) a blank solution with a mask substrate dissolved in diluent and (e) a blank solution of tissue dissolved in diluent were prepared and analyzed. When comparing these chromatograms (Figure 2), no additional peaks were observed at the elution times of the analytes.

#### 2.2.3. Linearity, LOD and LOQ

Linearity is assessed by plotting the signal (Area Under the Curve, AUC) against analyte concentration. From the data obtained, standard calibration curves were generated using the least squares method (Table 3). All were linear as their correlation coefficient (R^2^) was greater than 0.999.

The limit of detection (LOD) describes the smallest amount of analyte that can be detected, while the limit of quantification refers to the amount that can be accurately quantified. Under present experimental conditions, these values were calculated (Table 2) based on the following equations:

$$LOD=3.3\;\times \;\frac{\sigma }{S}\;\;\;\mathrm{and}\;\;\;LOQ=10\;\times \;\frac{\sigma }{S}$$

where

σ: standard deviation of the response, representing the noise level;

S: the slope of the calibration curve, used to convert signal to concentration.

#### 2.2.4. Precision

Intra-day precision was calculated by analyzing samples at three concentration levels (low, medium and high) on the same day with three replicates (n = 3). The results were evaluated based on the relative standard deviation (%RSD) values, which were found to be <2.0% (Table 4). Inter-day precision (between days) was studied by analyzing samples (n = 3) at three concentration levels on three consecutive days.

#### 2.2.5. Accuracy

To verify the accuracy of the proposed analytical method, three standard solutions, corresponding to three concentration levels (high, medium and low) of APIs, were prepared and determined based on their calibration curve (Table 3). Their % Recovery values ranged from 98.2 to 103.1% (%RSD < 1.2).

#### 2.2.6. Robustness

Robustness indicates the reliability of an analysis with respect to small possible variations (±1%) in the method parameters. To assess robustness, small modifications were introduced to the critical parameters, such as temperature, λmax, etc. (Table 5). Examining the %RSD of the AUC and Tf (tailing factor) values, it was observed that changes in the injection volume of the samples affected the results. This was partly expected, as substances with high detection sensitivity (high values of the molecular absorption coefficient) significantly affect their AUC values, even with small changes in their concentration. Therefore, to ensure the validity of the results, it is recommended to inject a standard solution between two or three samples.

### 2.3. Mask Preparation

According to the literature, pharmaceutical/cosmetic formulations that are commercially available usually contain benzoyl peroxide at concentrations of 2.5–10%, curcumin 0.1–0.5%, salicylic acid 0.5–2%, rosmarinic acid 0.2–5% and resveratrol 0.5–1% [1,44]. In the peel-out face mask prepared in our lab, all active ingredients were incorporated at a concentration of 0.5% (equivalent to 50 mg API/10 g mask), except for BENZ, for which the concentration was 2.5% (250 mg BENZ/10 g mask).

Peel-out masks solidify upon application, forming a single, relatively solid film that is removed by peeling. They usually contain an ingredient (such as alcohol), which when evaporated causes the mask to adhere to the skin, as well as a synthetic polymer to retain water and provide elasticity and stability to its texture. The most commonly used ingredients can be of natural (coconut, apple, orange extracts, green clay, gelatin and honey) or chemical (glycerin and polyvinyl alcohol) origin [16,45].

In the present study, clay, gelatin, honey, water and an ethanol/PEG (3:1) mixture were used as excipients. Green clay creates a protective film that absorbs toxins, bacteria and viruses, tightens pores, removes sebum and oil, improves blood circulation and gently exfoliates, leaving the skin soft [46]. Gelatin is a biopolymer derived from collagen and protein and provides cohesion to the substrate [47]. Finally, honey presents a strong moisturizing and emollient effect, as well as possessing antimicrobial, antiseptic and healing properties [48,49]. The ethanol/PEG (3:1) mixture has a dual role: it helps to incorporate the active ingredients into the mask and acts as a preservative.

In order to prepare a clay mask that is not too watery, is compact, soft in texture and spreads easily, a series of different proportions of excipients were investigated to find the most suitable composition. In detail, the following were studied: gelatin (which gives a sticky, solid texture) in a proportion of 0.75 to 6.6% *w*/*w*, water (which makes the composition more watery) from 20 to 33% *w*/*w*, EtOH/PEG 3:1 *v*/*v* from 10 to 20% *w*/*w*, clay from 33 to 55% *w*/*w* and honey from 7 to 10%. Considering the specifications of the new proposed face mask, a series of different excipient compositions were prepared, of which the following was considered optimal: APIs 4.5%, EtOH/PEG 3:1 13.5%, water 22.3%, gelatin 0.76%, honey 8.9% and clay 50% *w*/*w*.

Therefore, for the preparation of 10 g of face mask, the following prescription was followed: Quantities of 50.0 mg RES, CUR, SAL and ROS, and 250 mg BENZ, accurately weighed, were transferred to a suitable container (mortar) containing 1.34 g of ethanol/PEG (3:1) and the total mixture was manually homogenized with a pestle (container A). In a separate glass beaker, which was placed in a water bath (50 °C), 2.23 g of water was initially added and then successively, and under continuous stirring, 0.076 g of gelatin and 0.89 g of honey were also added. The mixture was gradually transferred to container A and, after being mixed well, the green clay was added (Supplementary Figure S3).

### 2.4. Fourier-Transformed Infrared Spectroscopy (FTIR)

FTIR analysis was conducted on all the raw materials and the prepared mask, in order to ensure the compatibility of their coexistence. The results are shown in Supplementary Figure S4. Clay presents a broad peak around 3000 cm^−1^, attributed to O–H stretching vibrations [50]. Gelatin presents a significant band (Amide I) at 1627 cm^−1^ [51], while honey has a broad peak at 3500–3000 cm^−1^ due to water content and a broad peak at 1160–930 cm^−1^ is attributed to C–O and C–C skeletal stretching vibrations of carbohydrates [52]. The APIs also present characteristic peaks. Specifically, BENZ has a peak at 1757 cm^−1^ attributed to C=O vibration [53], SAL has a C=O stretch of the carboxylic acid group at 1654 cm^−1^ [54], RES has –OH stretching bands at 3300 cm^−1^ owing to phenolic hydroxyl groups [55], ROS has two peaks at 1637 and 1552 cm^−1^ associated with the bending of the N-H bonds of amide I and amide II [56] and CUR has a peak at 1634 cm^−1^ corresponding to the C=O stretching β-diketone group. All these peaks are absent from the mask’s spectrum, indicating that all the APIs are molecularly dispersed inside the matrix or below the detection limit of the instrument.

### 2.5. Samples Pretreatment in Mask

#### 2.5.1. Experimental Design Methodology

**Figure 3:**
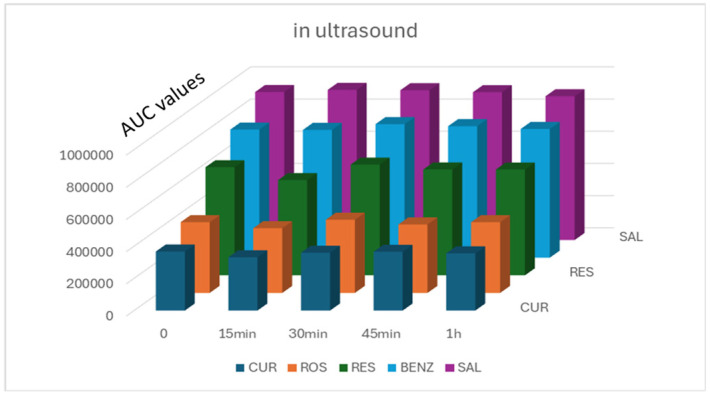
Stability study of analytes subjected to sonication, at room temperature (diluent: MeOH-ACN with 0.1% TFA 2:1 *v*/*v*).

A solid/liquid extraction method was applied to recover the five active ingredients from the clay mask substrate (100 mg). Since this is a process influenced by multiple variables, it was considered appropriate to use an experimental design methodology (Design–Expert 11). The extraction solvents (methanol and/or acetonitrile), their concentration ratio, the sonication time and the freezing time were examined as factors (Table 6, Supplementary Table S2). Sonication, at room temperature, for up to one hour (Figure 3) did not destroy the samples and contributed to the quantitative recovery of the analytes. On the contrary, the treatment of the samples by heating was not used because the APIs were unstable at 37 °C.

Therefore, in the present experimental conditions, using the D-optimal methodology (18 experiments), the combination of a solvent mixture (acetonitrile–methanol) was correlated with two factors related to the sample treatment (sonication time and freezing time).

The significance of these factors was assessed by analysis of variance (ANOVA), taking into account the values of specific statistical parameters (Table 7, Supplementary Table S3). The F value expresses the ratio of systematic dispersion due to a factor to the random unsystematic dispersion (F should > 1). The “R-squared” and the “adjusted R-squared” are intended to have similar values, as this ideal case means that 100% of the observed deviations can be explained by the model. According to the results, the composition of the extraction medium is significant and affects the recoveries of ROS, SAL and RES.

In addition, the freezing time (factor D) was considered important only for salicylic acid and not for the other components. Essentially, freezing is used indirectly to ensure the purity of the samples (precipitation of fatty impurities due to very low temperature) and not to improve their recovery rate. Thus, when determining the limits of the model factors, factor D was set to tend towards to the maximum possible value.

**Figure 4:**
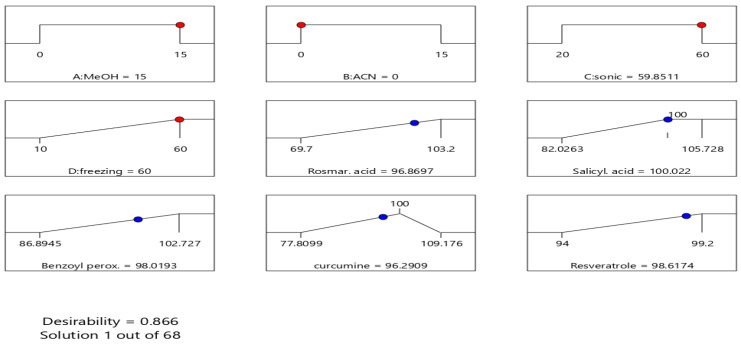
Diagrams depicting the optimal suggested values for the factors and the expected values of the responses.

The good predictive ability of the model is also highlighted by the linear correlation between the predicted and actual values depicted in the diagrams in Supplementary Figure S5. To find the optimal conditions for conducting the extraction experiment, the desirability function [57] was used and the process was modeled as follows (Figure 4):

In total, 100 mg of mask sample was quantitatively transferred to 15 mL MeOH, sonicated for 60 min (in water bath cooling), frozen for 60 min and centrifuged (5000 rpm for 5 min). Then, 500 μL of the supernatant was diluted with 250 μL ACN with 0.1% TFA and analyzed by HPLC.

#### 2.5.2. Validity of the Extraction

To validate the method, the same extraction procedure was repeated on five 100.0 mg mask samples. Each sample was spiked with 200 μL of APIs (ROS = 0.5 mg, RES = 0.5 mg, SAL = 0.5 mg, CUR = 0.5 mg and BENZ = 2.5 mg in MeOH), which, after being homogenized with the substrate, was gently evaporated to remove the solvent (30–45 min with a nitrogen evaporator, at room temperature). Then, based on the proposed extraction procedure, the samples were analyzed and quantified using an equivalent standard reference solution. The average recovery values of the analytes were found (Table 8) to be close to the predicted targets (ROS: 98.2% with %RSD = 1.0, RES: 99.6% with %RSD = 2.4, SAL: 99.9% with %RSD = 1.3, CUR: 99.4% with %RSD = 2.4, and και ΒΕΝΖ: 99.1% with %RSD = 1.6.

### 2.6. Transdermal Permeability

#### 2.6.1. Preliminary Studies

When a new cosmetic/therapeutic skin preparation is proposed, three points should be considered, in detail, to achieve the desired results. First, the substrate should release the APIs within 20–30 min, which is the usual application time of the mask. It should also be studied whether the drugs are sensitive to body temperature, i.e., 36–37 °C. Finally, it should be ensured that the active ingredients remain in the upper layers of the epidermis and not in the general bloodstream.

In the present study, the simulation of the transdermal permeability of five APIs was tested using the Franz cell diffusion apparatus (37 °C). To perform the experiments, two different substrates were tested, the mask under consideration and an aqueous suspension of water with 10% ethanol/PEG 3:1. The suspension was prepared containing equivalent amounts of active ingredients to those of the mask and was used as reference preparation, since there was no equivalent on the market. Each formulation was studied in triplicate using, as permeability barriers, a cellulose membrane (in vitro), a rat tissue and a human skin (ex vivo). Since the APIs initially encountered the donor surface (37 °C) and then passed into the acceptor compartment and dissolved in PBS (37 °C), it was important to study their stability in these two different conditions.

**Figure 5:**
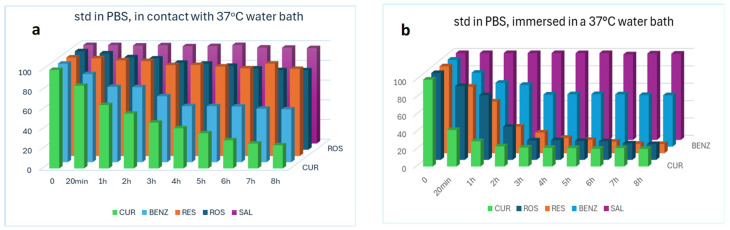
Stability study of analytes diluted in PBS solution, which was (**a**) in contact with a water bath at 37 °C and (**b**) immersed in the water bath at 37 °C.

Therefore, a standard mixed solution of SAL, RES, ROS, BENZ and CUR was prepared and placed on the surface of a water bath (37 °C) and another similar one was immersed in the same water bath at 37 °C. At regular intervals, samples were taken from these two solutions and analyzed. According to the results presented in Figure 5a,b only SAL was generally stable at 37 °C while the remaining APIs were quite stable in the first solution and only in the first 20 min.

**Figure 6:**
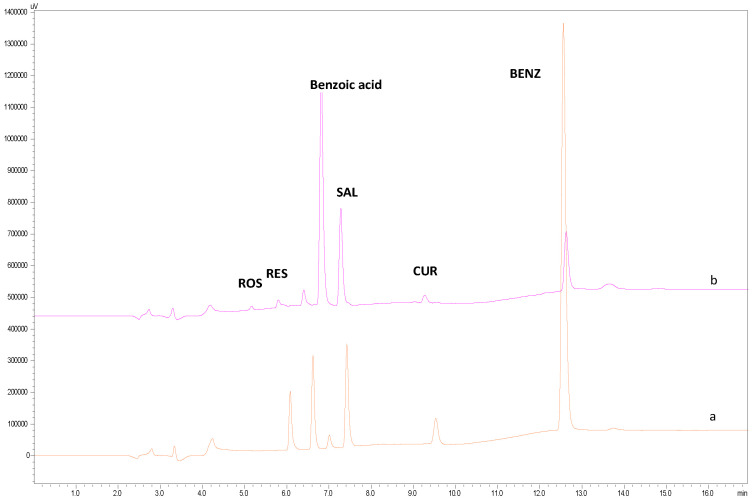
Standard solution subjected to heating at 37 °C (a) at 0 h, (b) after 2 h.

It was also found that in the second solution the destruction rate of CUR, ROS, RES and BENZ was much more significant. Another remarkable observation was that BENZ hydrolyzed over time to benzoic acid, giving a second chromatographic peak at 7.0 min (Figure 6). A very small percentage of this hydrolysis (0.95%) occurs even at time t = 0, which is probably due to the initial purity of BENZ (raw material).

In general, benzoic acid is also used in cosmetics as a preservative to prevent bacterial growth and its mechanism of action is directly related to that of BENZ. More specifically, the antimicrobial activity of BENZ is due to its ability to oxidize proteins of bacterial cell membranes. Oxidation occurs in the stratum corneum after BENZ penetrates and enters the hair follicle, where it is broken down into benzoic acid and hydrogen peroxide. Benzoic acid in turn produces free radicals, which have oxidizing properties [6,58]. Therefore, due to their synergistic action, the sum of both substances is recorded in Figure 5 as BENZ.

#### 2.6.2. Samples Pretreatment

For the analysis of the samples collected from Franz cells, special attention was paid to the following: (a) PBS salts should be removed, in order to maintain the good condition of the column and the HPLC apparatus (protection from high back pressure), (b) water evaporation should be performed gently, using the lyophilization method to avoid thermal decomposition of the active ingredients, and (c) methanol should be used as the optimal solvent for the reconstitution of the analytes, due to its high solubilizing capacity.

For the reliability of the proposed method, five mask samples (100 mg) containing 50 μg of ROS, RES, SAL and CUR and 250 μg of BENZ should be tested separately. This amount corresponds to 10% of the stated amount of APIs (0.5% *w*/*w* for ROS, RES, SAL, CUR and 2.5% for BENZ), which theoretically passed from the donor to the acceptor compartment (V = 5 mL PBS). Of course, since in this case our purpose was to check the recovery of samples taken from the receiver compartment and not from the mask, these quantities were calculated and adjusted accordingly to the samples under consideration.

Thus, each sample tested contains 5 μg of ROS, RES, SAL and CUR and 25 μg of BENZ dissolved in 500 μL of PBS. Every sample was frozen for 5 h and then lyophilized (24 h) to remove the aqueous phase. After adding 200 μL of methanol to the solid residue (API and PBS), the sample was vortexed (5 min), sonicated (20 min), frozen (2 h) and centrifuged (10 min, 10 K × 1000 rpm). Then, 100 μL of the supernatant was carefully removed, transferred to a vial insert and, after being diluted with 50 μL of 0.1% ACN/TFA, analyzed by HPLC. The samples were quantified based on a standard solution with a final concentration of 16.6 μg/mL ROS, RES, SAL and CUR and 83.3 μg/mL BENZ. The satisfactory values for both % Recovery and %RSD confirm the reliability of the method (Table 9).

#### 2.6.3. Evaluation of Permeability Results

After the completion of the permeability experiments, with Franz cells, the behavior of the APIs was recorded and evaluated. To draw reliable conclusions, certain physicochemical properties of the active ingredients were taken into account, such as their lipophilicity index (distribution coefficient log P), their size (MW) and their solubility in water, which are directly related to the phenomenon (Supplementary Table S1). Of course, under no circumstances should their rate of destruction in PBS (37 °C) be overlooked.

**Figure 7:**
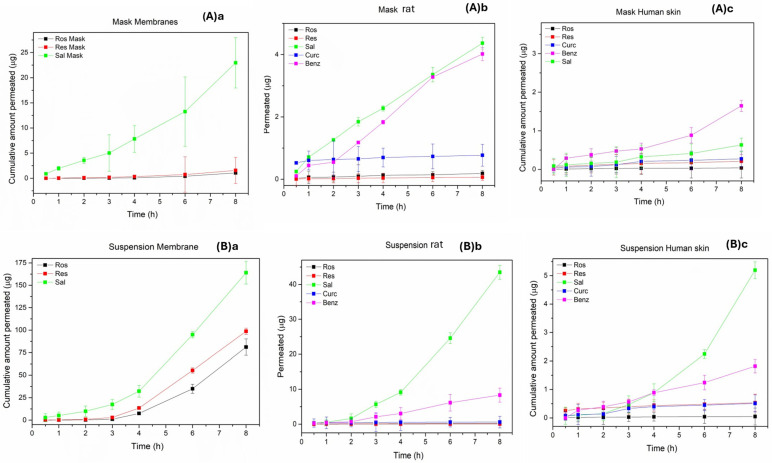
Cumulative permeability amount of APIs using as carrier (**A**) face mask and (**B**) suspension in (**a**) cellulose membrane, (**b**) rat tissue and (**c**) human skin.

The profile of the cumulative amount of each compound that penetrated the acceptor chamber is shown in Figure 7, while the steady-state flux Jss and the Papp coefficient are presented in Table 10. The amount of APIs added to the donor (Franz cells) and the corresponding ones that were trapped in the membrane or transferred to the receiver are shown in Table 11.

An overall observation was that the mask, compared to the suspension, is able to retain higher percentages of APIs in the donor compartment and therefore could have a better performance for local treatment. Similarly, in all cases, the substance with the highest penetration ability was SAL, which has the lowest molecular weight, the highest solubility in water, high polarity and is not destroyed at 37 °C. Comparing the barriers with each other, we would say that in human tissue, all the analytes penetrated less than in the cellulose membrane and rat skin. The cellulose membrane, from a structural point of view, presents a relatively polar surface and seems to favor the penetration of only the three substances, ROS, RES and SAL. All three have good solubility in water and relatively small molecular weights. Also, two of them, ROS and SAL, when diluted in PBS (pH 7–7.4), are in their ionized form and are therefore more polar than the others.

Another notable observation was that BENZ, in all cases (except 0.5 h), was detected in the receptor compartment as benzoic acid. This is largely due to its high hydrolysis rate (in PBS at 37 °C) and its low solubility in water.

Finally, based on the Papp values of the APIs, we can say that the barrier that most closely mimics human tissue was that of the rat. Indeed, the ranking of the Papp values of the APIs, in descending order, was the same in the two tissues (SAL, BENZ, CUR-ROS and RES), while in the cellulose membrane it was different (SAL, RES and ROS while CUR and BENZ were not detected). To verify the reliability of the results, parallel control tests were performed on the APIs (loading and found values) on both the mask samples and the suspensions (Table 11). In addition, Table 11 presents the total amounts of analytes that remained (a) in the cellulose membrane, (b) in the rat tissue and (c) in the human skin after the completion of the experiments (8 h), as well as their maximum amounts determined in the receptor compartment. The results could be considered to be in reasonable correlation with each other if the high % decomposition rate of the analytes is taken seriously into account. More specifically, after 8 h, SAL remains stable in PBS solution (37 °C) at approximately 96%, BENZ at 60%, RES at 10%, ROS at 18% and CUR at 20% (Figure 5b).

## 3. Materials and Methods

### 3.1. Apparatus

#### 3.1.1. Instrumentation

Separation was carried out with a Shimadzu Model LC-20AD pumps (Shimadzu, Kyoto, Japan), using a gradient elution system and a mobile phase composed of two solvents (A: H_2_O with 0.1% TFA-ACN with 0.1% TFA, 85:15 *v*/*v*; B: 100% ACN with 0.1% TFA). A UV diode array detector was set at appropriate wavelengths (320, 234 and 400 nm). In total, 20 μL of each sample was injected with a SIL-20AD auto-sampler and the analytes were separated on a HS C18 (25 cm × 4.6 mm I.D.) Supelco Discovery (Supelco Inc., Bellefonte, PA, USA) column packed with 5 μm sized particles (thermostated at 40 °C). All experiments were proceeding in duplicate with a flow rate of 1 mL/min. The chromatographic peaks were recorded and elaborated through Shimadzu LC Solution software 2020.

Fourier-Transformed Infrared Spectroscopy (FTIR) analysis was conducted to investigate the chemical interactions between the APIs and the other components. For this purpose, an IR Prestige-21 instrument (Shimadzu, Kyoto, Japan) was employed, and FTIR spectra of all the raw materials and the prepared mask were acquired. The analysis was conducted in the range of 600–4000 cm^−1^ with a resolution of 2 cm^−1^ and the results were plotted against Transmittance (%).

Lyophilization was conducted using a VirTis Benchtop SLC freeze dryer (VirTis, Salem, India), operating at 100 bar and at −70 °C. The drying process was conducted for 6 h and after that the samples were immediately analyzed using HPLC.

#### 3.1.2. Permeability Studies: Franz Cells

The permeability profiles of RES, CUR, SAL, ROS and BENZ, incorporated into the face mask or suspension, were studied using a vertical Franz cell diffusion system. The cells consist of two compartments, a donor (upper chamber) and an acceptor (lower chamber). Between the two chambers, three different barriers (active surface area 2 cm^2^) were fitted: (a) cellulose membrane, (b) skin collected from the dorsal region of albino wistar rats and depilated and (c) human tissue collected from the lateral abdominal region. To carry out the experiments, the donor compartment was coated either uniformly with 100 mg of mask or with 100 µL of suspension, while the receiver was filled with 5 mL of degassed PBS, pH 7.4 (at 37 °C), under sink conditions and with continuous stirring (90 rpm). At regular time intervals (0.5, 1, 2, 3, 4, 6 and 8 h), a 0.5 mL sample volume was taken from the receiver compartment and replaced with fresh pre-warmed PBS.

Experiments were repeated in triplicate for both mask and suspensions, while four additional samples were also examined separately as blanks. Two blanks containing only the substrate (mask and suspension) and two samples of mask and suspension were analyzed directly without being subjected to Franz cells (loaded amounts).

Samples, after lyophilization and appropriate processing, were analyzed by HPLC. Each sample was analyzed in duplicate and its cumulative release over time was plotted. The steady-state flux (Jss) was determined by plotting the permeation of API per unit area (μg/cm^2^) against time (h) and calculating the slope of the linear portion of the resulting line. The following equation was applied to calculate the apparent permeability coefficient (Paap).

Papp = Jss/Cd (cm/h),

where Cd is the initial concentration of the drug in the donor compartment and Jss is the steady state flux.

### 3.2. Reagents and Solutions

All solvents acetonitrile (CH_3_CN, or ACN Honeywell), methanol (CH_3_OH or MeOH, Chem-Lab, Petaling Jaya, Malaysia), ethanol (CH_3_CH_2_OH, EtOH) (Chem-Lab), trifluoroacetic acid (CF_3_COOH, TFA, Sigma-Aldrich, Saint Louis, MO, USA), formic acid (HCOOH, FA, Sigma-Aldrich) and H_2_O (Adrona Q-Front Water Purification System) were of HPLC grade, appropriate for chromatographic analysis.

Salicylic acid (SAL, C_7_H_6_O_3_), benzoyl peroxide (BENZ, C_14_H_10_O_4_), rosmarinic acid (ROS, C_18_H_16_O_8_) and curcumin (CUR, C_21_H_20_O_6_) were purchased from (Sigma-Aldrich) while resveratrol (RES, C_14_H_12_O_3_) from TCI Chemicals. The gelatine, honey and green clay used for the preparation of the face mask were of natural origin and were obtained from a local shop in Thessaloniki.

For the preparation of PBS, solutions at pH 7.4 are required: NaCl 8 g/L; KCl 0.2 g/L; Na_2_HPO_4_ 1.44 g/L; and KH_2_PO_4_ 0.24 g/L.

### 3.3. Standard Solutions and Sample Pretreatment

#### 3.3.1. Standard Solutions

For the preparation of standard solutions, approximately 4 to 5 mg of each analyte was accurately weighed into separate 10 mL volumetric flasks, which were filled to the mark with methanol (stock standard solutions). Different volumes of the initial solutions were transferred to an intermediate 100 mL volumetric flask, which was filled to the mark with diluent (MeOH-ACN with 0.1% TFA 2:1 *v*/*v*). Finally, with different dilutions, in 10 mL volumetric flasks, a series of six standard solutions was prepared (diluent: MeOH-ACN with 0.1% TFA 2:1 *v*/*v*).

#### 3.3.2. Face Mask Extraction

To a 100 mg mask, which was accurately weighed into a 25 mL volumetric flask, 15 mL of MeOH was added and placed in a cooled ultrasonic bath for 60 min. The sample was then frozen for 60 min and centrifuged at 5000 rpm for 5 min. A total of 500 μL of the supernatant was taken and mixed with 250 μL of ACN/0.1% TFA and analyzed by HPLC.

#### 3.3.3. Sample Processing in In Vitro/Ex Vivo Permeability Experiments

The samples resulting from the in vitro/ex vivo experiments were collected (500 μL) at specified time intervals and, after being appropriately processed, were analyzed by HPLC. In detail, the collected samples were subjected to lyophilization (24 h) to remove the aqueous phase. To the solid residue obtained after lyophilization, 200 μL of methanol was added and the sample was then vortexed (5 min), sonicated (20 min), frozen (2 h) and centrifuged (10 min, 10 K × 1000 rpm). Finally, 100 μL of the supernatant was carefully removed, transferred to a vial insert and, after being diluted with 50 μL of ACN/TFA 0.1%, analyzed by HPLC.

Similarly, after the Franz cell experiments were completed, the membranes and tissues, which were coated with 100 mg mask or 100 μL suspension, were collected separately and subjected to the following procedure (Section 2.5.2). Each sample (substrate) was quantitatively transferred to a beaker to which 15 mL of methanol was added. This was followed by sonication (60 min) and freezing (60 min), and 1 mL of the supernatant sample was subjected to centrifugation (10 min). Then, 500 μL of the supernatant and 250 μL of ACN/TFA 0.1% were added to a vial that, after mixing (vortex 1 min), was analyzed by HPLC.

## 4. Conclusions

In the present study, a new cosmetic/therapeutic facial mask with synergistic anti-acne activity was created. Four of its active ingredients (salicylic acid, curcumin, rosmarinic acid and resveratrol) were incorporated into the composition at a concentration of 0.5% *w*/*w*, while benzoyl peroxide at 2.5% *w*/*w*. Clay, gelatin, honey, water and an ethanol/PEG mixture (3:1) were used as excipients.

Subsequently, a reverse-phase HPLC-DAD method was successfully developed and validated for the quantitative determination of the active ingredients in the mask (liquid/solid extraction) and in Franz cells (permeability studies). To conduct the study, the stability of the active ingredients was investigated during (a) the preparation of the mask, (b) the application of their extraction method and (c) the permeability study of the APIs using Franz cells. Based on the results, the analytes remain stable when dissolved in organic solvents (acetonitrile and methanol with or without TFA) or when subjected to sonication, but are unstable when dissolved in PBS at 37 °C. These conditions simulate body fluids and for the first 20 min (considered the required mask application time) the APIs remain almost stable, while after 8 h they are maintained at only 10% of RES, 18% of ROS, 20% of CUR, 60% of BENZ and 96% of SAL. The % Recovery values of BENZ, under the given conditions, were calculated as the sum of benzoyl peroxide and benzoic acid, which is its hydrolysis product.

Finally, the effectiveness of the facial mask formulation was evaluated, based on the ability of the active ingredients to penetrate the skin. Its performance was compared with a corresponding suspension formulation of equivalent activity. The experiments were conducted with Franz vertical diffusion cells on three different tissues (cellulose membrane, rat tissue and human skin). According to the results, the mask exhibits better behavior as only a very small percentage of the APIs penetrate the skin, while the rest remains on the surface, healing it. Regarding the choice of the membrane that most closely resembles human skin, between cellulose or rat tissue, it seems that the tissue clearly prevails.

## Figures and Tables

**Table 1:** The gradient elution program of the mobile phase.

Time (Min)	A%	B%	Flow (mL/Min)
0	100	0	1.0
5	40	60	1.0
7	40	60	1.0
9	20	80	1.0
14	20	80	1.0
15	100	0	1.0
20	run	stop	1.0

**Table 2:** System suitability parameters.

Name	Retention Time	Tailing Factor	K′	Resolution	Theoretical Plates	HETP × 103 USP
ROS	5.9	1.5	1.4	--	25,068	9.97
RES	6.4	1.4	1.6	3.9	31,327	7.98
SAL	7.2	1.3	1.9	2.7	32,309	7.74
CUR	9.3	1.2	2.7	11.3	37,360	6.69
BENZ	12.2	1.3	3.9	16.8	65,485	3.82

**Table 3:** Concentration limits and characteristics of the calibration curves of the five APIs.

Substances	Concentration Range (μg/mL)	Calibration Curves	Correlation Coefficient (r^2^)	LOD (μg/mL)	LOQ (μg/mL)
ROS	1.60–16.0	y = 58,698x + 1713.6	0.9998	0.267	0.810
RES	0.37–15.10	y = 160,221x + 1322.3	0.9999	0.047	0.142
SAL	2.04–20.40	y = 14,367x + 5208.4	0.9994	0.636	1.925
CUR	0.92–9.20	y = 88,301x − 7886.7	0.9994	0.296	0.897
BENZ	1.35–22.5	y = 80,502x + 16,089	0.9991	0.083	0.251

**Table 4:** Intra- and inter-day precision and accuracy.

APIs	Intra-Day (n = 3)		Accuracy		Inter-Day Precision (n = 3)			
	Concentration	%RSD	% Recovery	%RSD	%RSD			
	(μg/mL)				1nd Day	2nd Day	3rd Day	Total (n = 12)
ROS	1.6	0.32	99.4	0.39	0.38	0.31	0.07	0.25
	8	0.15	101.1	0.01	0.20	0.14	0.12	0.20
	16	0.17	100.5	0.19	0.19	0.10	0.07	0.19
RES	0.376	0.47	100.5	0.33	0.33	0.09	0.36	0.45
	1.88	0.05	100.3	0.15	0.07	0.04	0.13	0.23
	15.1	0.17	100.0	0.01	0.19	0.10	0.08	0.14
SAL	2.04	0.44	98.2	1.2	0.04	0.47	0.43	0.42
	10.2	0.13	100.5	0.2	0.19	0.08	0.29	0.18
	20.4	0.15	100.2	0.23	0.22	0.02	0.10	0.14
CUR	0.92	0.52	101.6	0.74	0.78	0.07	0.45	0.47
	4.6	0.28	100.2	0.13	0.14	0.38	0.30	0.27
	9.22	0.23	101.3	0.03	0.03	0.32	0.05	0.27
BENZ	1.35	1.12	103.1	0.33	1.36	0.63	0.82	0.98
	4.5	0.41	99.1	0.97	0.48	0.15	0.16	0.43
	22.5	0.69	100.5	0.85	0.25	0.46	0.34	0.67

**Table 5:** Robust investigation.

	%RSD									
Parameters	AUC ROS	Tf ROS	AUC RES	Tf RES	AUC SAL	Tf SAL	AUC CUR	Tf CUR	AUC BENZ	Tf BENZ
Mob.phase %B	0.5	1.4	1.3	5.1	1.9	0	0.6	0.8	1.2	4.7
(84, 85, 86)										
Column (°C)	1.3	0.5	0.2	1.3	0.2	1.7	2	1.7	0.7	2.5
(39, 40, 41)										
λ max	2	0.5	3.3	0.5	2	0.5	2.9	0.9	1.1	0
Inj. volume (19, 20, 21)	4.6	2.1	4.9	3	4.5	0.5	4.8	0.8	1.4	0.8

**Table 6:** Description of the D-optimal experimental design model.

Study Type	Combined		Subtype	Randomized
Design Type	D-optimal		Runs	18
Design Model	Quadratic × Quadratic		Blocks	No Blocks
Build Time (ms)	104.00			
Mixture	Components	A (MeOH) + B (ACN) = 15		
Process	Factors	C Sonic (20–60 min)		
		D Freezing time (10–60 min)		

**Table 7:** ANOVA results of the suggested mixture/D-optimal model.

Parameters	ROS	SAL	RES
Std. Dev.	4.81	2.67	0.67
C.V. %	5.35	2.79	0.69
R^2^	0.705	0.85	0.85
Adjusted R^2^	0.697	0.77	0.82
Predicted R^2^	0.570	0.58	0.78
Adeq Precision	113.0	136.6	124.9

**Table 8:** Validity of the extraction (n = 5).

	% Recovery				
Samples	ROS	RES	SAL	CUR	BENZ
n_1_	98.1	102.1	99.9	101.2	100.6
n_2_	97.6	100.3	98.7	99.7	99.5
n_3_	99.9	101.4	98.9	101.1	99.8
n_4_	97.3	96.3	101.9	95.4	96.5
n_5_	98.0	98.1	100.0	99.8	99.0
%RSD	1.0	2.4	1.3	2.4	1.6

**Table 9:** Reliability of the method.

	ROS	RES	SAL	CUR	ΒΕΝΖ
	105.4	106.9	105.3	103.0	106.2
	96.0	94.7	94.8	90.6	91.1
	102.0	102.5	98.5	100.2	94.6
	101.8	94.2	99.5	95.5	96.8
	104.4	99.7	96.6	98.0	99.9
Mean	101.9	99.6	98.9	97.5	97.7
%RSD	3.2	4.8	3.6	4.3	5.2

**Table 10:** Steady-state flux Jss and Papp coefficient calculation ROS, RES, SAL, CUR and BENZ.

**Cell**	**J (μg cm^−2^ min^−1^) Rat Skin**				
	**ROS**	**RES**	**SAL**	**CUR**	**BENZ**
mask	0.0035 ± 0.005	0.00135 ± 0.00005	0.10185 ± 0.00485	0.0049 ± 0.0006	0.104 ± 0.0041
Suspension	0.0031 ± 0.003	0.00125 ± 0.00005	1.3394 ± 0.0233	0.0029 ± 0.0008	0.2145 ± 0.0372
**Cell**	**P_app_ (cm min^−1^) Rat Skin**				
	**ROS**	**RES**	**SAL**	**CUR**	**BENZ**
mask	6.80 ± 0.97	2.78 ± 0.103	153.16 ± 7.293	9.51 ± 1.17	42.10 ± 167
Suspension	6.26 ± 0.60	2.52 ± 0.1	2626.27 ± 45.69	5.47 ± 1.50	86.84 ± 15.06
**Cell**	**J (** **μ** **g cm^−2^ min^−1^) Membrane**				
	**ROS**	**RES**	**SAL**	**CUR**	**BENZ**
mask	0.03915 ± 0.002	0.0455 ± 0.0002	0.53335 ± 0.028		
Suspension	3.062 ± 0.05	3.261 ± 0.128	5.05895 ± 0.368		
**Cell**	**P_app_ (cm min^−1^) Membrane**				
	**ROS**	**RES**	**SAL**	**CUR**	**BENZ**
mask	76.02 ± 4.37	93.81 ± 0.41	802.03 ± 42.03		
Suspension	6185.85 ± 101.01	6587.57 ± 257.67	9919.51 ± 722.06		
**Cell**	**J (μg cm^−2^ min^−1^) Human**				
	**ROS**	**RES**	**SAL**	**CUR**	**BENZ**
mask	0.00285 ± 0.0022	0.00305 ± 0.00004	0.0135 ± 0.0002	0.00655 ± 0.00115	0.05225 ± 0.0008
Suspension	0.00075 ± 0.00005	0.00555 ± 0.00015	0.1777 ± 0.02	0.0117 ± 0.0004	0.04585 ± 0.0007
**Cell**	**P_app_ (cm min^−1^) Human**				
	**ROS**	**RES**	**SAL**	**CUR**	**BENZ**
mask	5.53 ± 4.17	6.29 ± 0.103	20.30 ± 0.30	12.72 ± 2.23	21.15 ± 0.30
Suspension	1.51 ± 0.10	11.21 ± 0.30	348.43 ± 40.20	22.08 ± 0.75	18.56 ± 0.26

**Table 11:** Amounts of each drug loaded and quantified.

**Rat Skin**		**Drugs Amounts in Suspension**					**Drugs Amounts in Mask**			
**Drug**	**Reference Sample**		**Donor**	**Acceptor**	**Membrane**	**Reference Sample**		**Donor**	**Acceptor**	**Membrane**
	**Loaded (μg)**	**Founded (μg)**	**Loaded Sample (μg)**	**Final Founded (μg)**	**Final Founded (μg)**	**Loaded (μg)**	**Founded (μg)**	**Loaded Sample (μg)**	**Final Founded (μg)**	**Final Founded (μg)**
ROS	495.0	510.0	510.0	0.12	228.6	515.0	512.0	512.0	0.13	162.7
RES	595.0	566.1	566.1	0.04	291.7	485.0	484.6	484.6	0.05	174.5
SAL	510.0	489.2	489.2	28.52	383.0	665.0	664.0	664.0	3.19	507.4
CUR	530.0	469.7	469.7	0.38	284.4	515.0	508.3	508.3	0.53	140.8
BENZ *	2470.0	2242.5	2242.5	6.82	1555.6	2470.0	2461.2	2461.2	3.27	1238.9
**Membrane**		**Drugs Amounts in Suspension**					**Drugs Amounts in Mask**			
**Drug**	**Reference Sample**		**Donor**	**Acceptor**	**Membrane**	**Reference Sample**		**Donor**	**Acceptor**	**Membrane**
	**Loaded (μg)**	**Founded (μg)**	**Loaded Sample (μg)**	**Final Founded (μg)**	**Final Founded (μg)**	**Loaded (μg)**	**Founded (μg)**	**Loaded Sample (μg)**	**Final Founded (μg)**	**Final Founded (μg)**
ROS	495.0	510.0	510.0	72.43	194.2	515.0	512.0	512.0	1.00	154.7
RES	595.0	566.1	566.1	88.74	158.5	485.0	484.6	484.6	1.35	117.2
SAL	510.0	489.2	489.2	145.46	261.7	665.0	664.0	664.0	19.16	554.9
CUR	530.0	469.7	469.72	0	239.1	515.0	508.3	508.3	0	153.6
BENZ *	2470.0	2242.5	2242.5	0	1456.1	2470.0	2461.2	2461.2	0	1044.2
**Human**		**Drugs Amounts in Suspension**					**Drugs Amounts in Mask**			
**Drug**	**Reference Sample**		**Donor**	**Acceptor**	**Membrane**	**Reference Sample**		**Donor**	**Acceptor**	**Membrane**
	**Loaded (μg)**	**Founded (μg)**	**Loaded Sample (μg)**	**Final Founded (μg)**	**Final Founded (μg)**	**Loaded (μg)**	**Founded (μg)**	**Loaded Sample (μg)**	**Final Founded (μg)**	**Final Founded (μg)**
ROS	495.0	510.0	510.0	0	297.2	515.0	512.0	512.0	0.00	171.7
RES	595.0	566.1	566.1	0.31	363.8	485.0	484.6	484.6	0.14	156.6
SAL	510.0	489.2	489.2	4.64	327.8	665.0	664.0	664.0	0.49	525.0
CUR	530.0	469.7	469.72	0.36	255.7	515.0	508.3	508.3	0.19	114.0
BENZ *	2470.0	2242.5	2242.5	1.52	1587.4	2470.0	2461.2	2461.2	1.35	1015.6

* ΒΕΝΖ is the sum of benzoyl peroxide and benzoic acid.

## Data Availability

The raw data supporting the conclusions of this article can be made available by the authors upon reasonable request.

## Supplementary Materials

The following supporting information can be downloaded at: https://www.mdpi.com/article/10.3390/molecules30224474/s1. Figure S1. Chemical structure of the five analytes. Table S1: Physicochemical properties of the analytes. Figure S2: UV spectra of ROS, CUR, BENZ, RES and SAL. Figure S3: Green clay face mask for acne treatment. Figure S4: FTIR spectra of raw materials. Table S2: Sample processing conditions and % Recovery results. Table S3: Optimal fitting models for the selected responses (Analysis of Variance). Figure S5: Predicted vs. actual values of ROS, SAL and RESV.
