# Supplementary material for: Development and Validation of an HPLC-DAD Method for the Quantitative Determination of Benzoyl Peroxide, Curcumin, Rosmarinic Acid, Resveratrol and Salicylic Acid in a Face Mask—In Vitro/Ex Vivo Permeability Study
Source: Molecules. 2025 Nov 19;30(22):4474. doi: 10.3390/molecules30224474 (PMC12655397; doi:10.3390/molecules30224474)
Supplement: Supplementary file 1 [file molecules-30-04474-s001.zip › molecules-3936310-supplementary.pdf]

Supplementary

Development and Validation of an HPLC-DAD Method for the Quantitative Determination of Benzoyl Peroxide, Curcumin, Rosmarinic Acid, Resveratrol and Salicylic Acid in a Face Mask—In Vitro/Ex Vivo Permeability Study

Sofia Almpani [1], Maria Mitsiou [1], Paraskevi Kyriaki Monou [2] and Catherine K. Markopoulou [1,*]

[1] Laboratory of Pharmaceutical Analysis, Department of Pharmacy, Aristotle University of Thessaloniki, 54124 Thessaloniki, Greece
[2] Laboratory of Pharmaceutical Technology, Department of Pharmacy, Aristotle University of Thessaloniki, 54124 Thessaloniki, Greece

* Correspondence: amarkopo@pharm.auth.gr; Tel.: +30-2310-997665

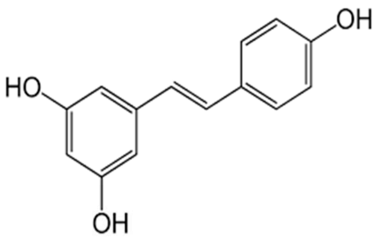

Resveratrol (RES)

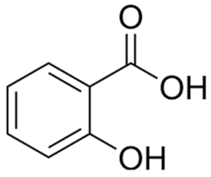

Salicylic acid (SAL)

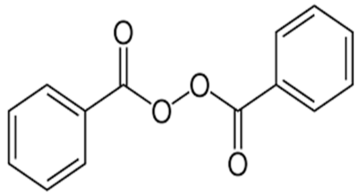

Benzoic peroxide (BENZ)

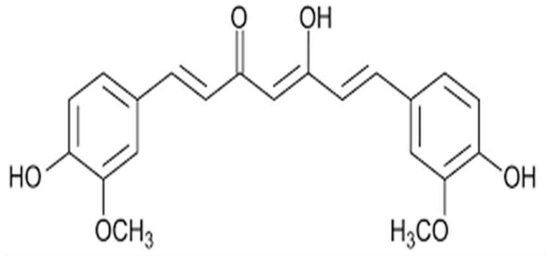

Curcumin (CUR)

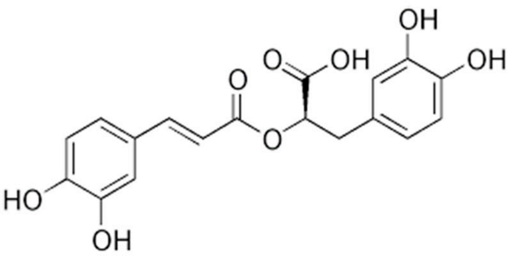

Rosmarinic acid (ROS)

Figure S1. Chemical structure of the five analytes

Table S1. Physicochemical properties of the analytes

	MW (g/mol)	logS	LogP	PSA (Å2)	Solubility in Water	pKacidic
ROS	360.3	-4	2.4	145	1-24 mg/mL	4.50
RES	228.24	-3.5	3.1	60.7	0.03 mg/mL	9.20
SAL	138.12	-1.09	2.26	57.5	1.24-77.79 mg/mL	2.97
CUR	368.4	very low	3.2	93.1	0.0111 mg/mL	7.8-8.5
BENZ	242.23	-3.9	3.5	52.6	0.35 mg/L	-

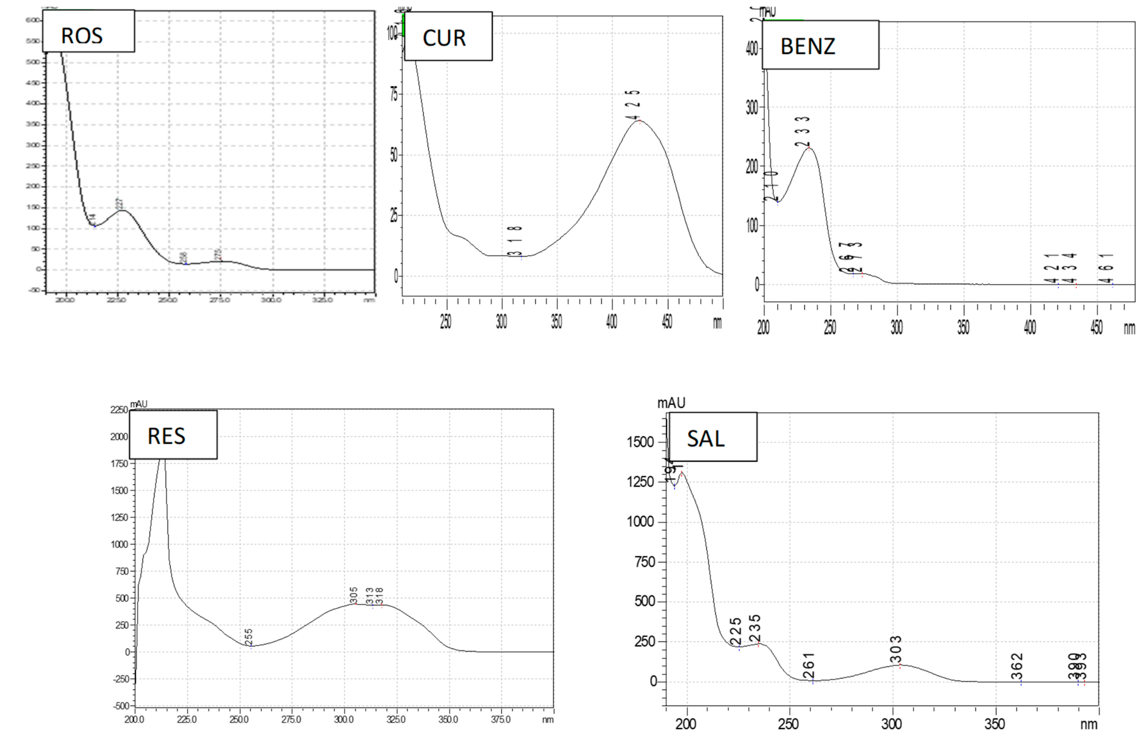

Figure S2. UV spectra of ROS, CUR, BENZ, RES and SAL

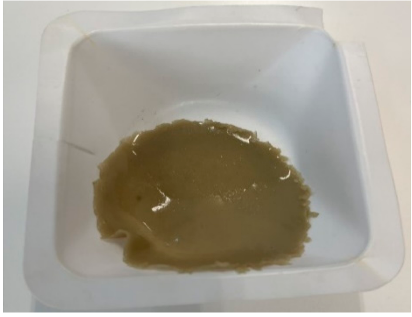

Figure S3. Green clay face mask for acne treatment

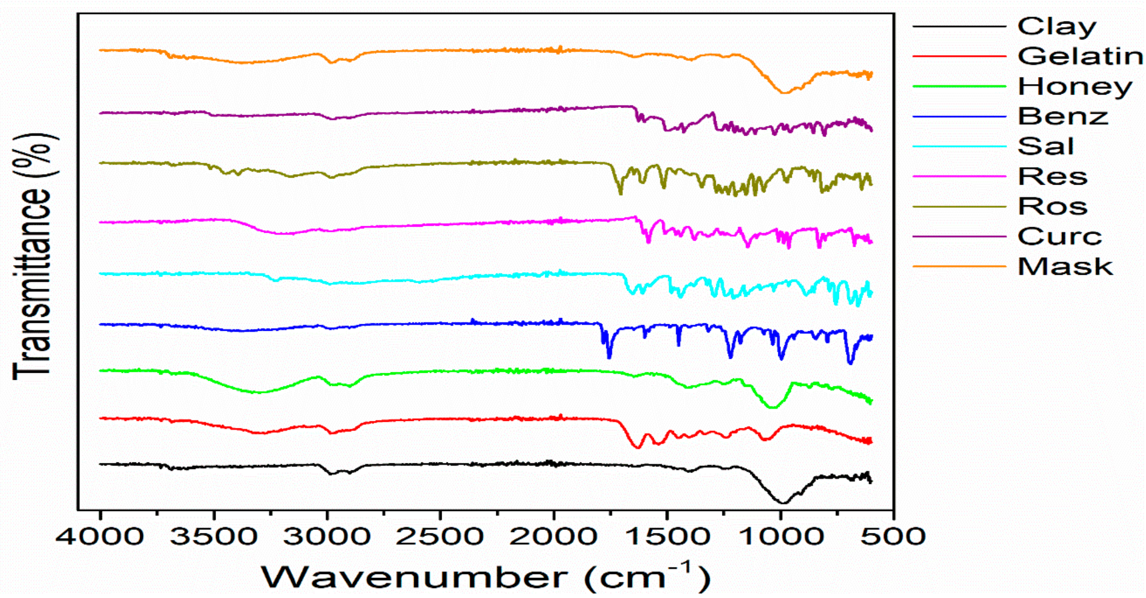

Figure S4. FTIR spectra of raw materials

Table S2. Sample processing conditions and %recovery results

	**Component 1**	**Component 2**	**Factor 1**	**Factor 2**	**Responses (%Recovery)**				
**Run**	**A:MeOH ml**	**B:ACN ml**	**C:sonic min**	**D:freezing min**	**ROS**	**SAL**	**BENZ**	**CUR**	**RES**
1	6.9	8.1	32.8	60.0	98.5	98.3	101.4	109.2	99.0
2	0.0	15.0	37.4	31.3	81.8	95.0	95.2	97.7	96.0
3	7.1	7.9	60.0	10.0	75.0	89.9	89.0	77.8	97.9
4	7.1	7.9	20.0	45.8	69.7	94.5	86.9	79.1	98.0
5	15.0	0.0	20.0	10.0	103.2	82.0	94.9	98.9	98.2
6	0.0	15.0	60.0	10.0	85.995	87.5	98.1	99.0	95.3
7	15.0	0.0	20.0	60.0	86.9	97.3	98.9	91.4	99.2
**8**	**0.0**	**15.0**	20.0	60.0	90.3	105.7	98.2	105.7	95.8
9	15.0	0.0	36.6	32.5	95.9	94.3	97.4	96.1	98.7
10	15.0	0.0	60.0	10.0	100.0	100.6	102.1	107.0	96.7

11	15.0	0.0	60.0	47.5	99.7	100.9	102.7	97.8	98.3
12	0.0	15.0	60.0	44.5	89.3	96.9	98.4	98.4	96.7
13	6.9	8.1	60.0	60.0	87.2	99.7	100.7	104.5	98.1
14	0.0	15.0	20.0	10.0	89.7	98.4	98.5	97.4	95.9
15	6.9	8.1	42.6	31.9	95.6	99.9	100.8	104.1	99.5
16	7.0	8.0	20.0	10.0	85.9	90.0	92.8	89.6	99.0
17	0.0	15.0	48.0	60.0	88.4	98.1	97.1	96.4	96.5
18	15.0	0.0	46.0	60.0	95.5	95.7	97.9	95.3	98.6

.

Table S3. Optimal fitting models for the selected responses (Analysis of Variance)

**Response 1: ROS**		
**Cubic (mixture) x Mean (process model)**		
**Source**	**F-value**	**p-value**
**Model**	14.01	0.0002
(1)Linear Mixture	11.73	0.0041
AB	28.12	0.0001
AB(A-B)	22.79	0.0003
**Response 2: SAL**		
**Reduced Linear (mixture) x 2FI (process model)**		
**Source**	**F-value**	**p-value**
**Model**	10.64	0.0005
(1)Linear Mixture	1.40	0.2625
AC	20.09	0.0009
AD	7.40	0.0199
BC	12.28	0.0049
BD	13.74	0.0035
ACD	5.76	0.0353
**Response 5: RES**		
**Quadratic (mixture) x Mean (process model)**		
**Source**	**F-value**	**p-value**
**Model**	41.45	< 0.0001
(1)Linear Mixture	76.47	< 0.0001
AB	6.44	0.0228

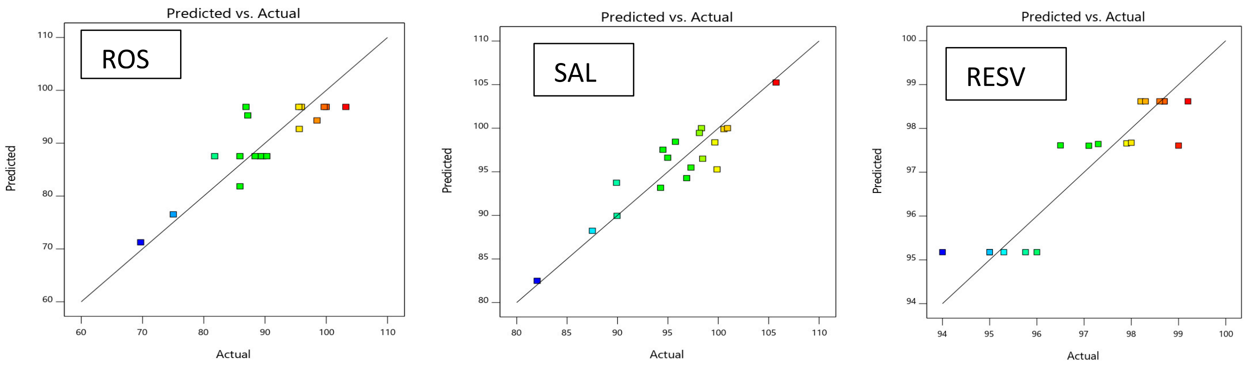

Figure S5. Predicted vs actual values of ROS, SAL and RESV.
